# Novel *In Vivo* Imaging Analysis of an Inner Ear Drug Delivery System in Mice: Comparison of Inner Ear Drug Concentrations over Time after Transtympanic and Systemic Injections

**DOI:** 10.1371/journal.pone.0048480

**Published:** 2012-12-12

**Authors:** Sho Kanzaki, Masato Fujioka, Akimasa Yasuda, Shinsuke Shibata, Masaya Nakamura, Hirotaka James Okano, Kaoru Ogawa, Hideyuki Okano

**Affiliations:** 1 Department of Otolaryngology Head and Neck Surgery, Keio University, Shinjuku-ku, Tokyo, Japan; 2 Department of Orthopedics, School of Medicine, Keio University, Shinjuku-ku, Tokyo, Japan; 3 Department of Physiology, School of Medicine, Keio University, Shinjuku-ku, Tokyo, Japan; Massachusetts Eye & Ear Infirmary, Harvard Medical School, United States of America

## Abstract

**Objective:**

Systemic steroid injections are used to treat idiopathic sudden-onset sensorineural hearing loss (ISSHL) and some inner ear disorders. Recent studies show that transtympanic (TT) steroid injections are effective for treating ISSHL. As *in vivo* monitoring of drug delivery dynamics for inner ear is lacking, its time course and dispersion of drugs is unknown. Here, we used a new *in vivo* imaging system to monitor drug delivery in live mice and to compare drug concentrations over time after TT and systemic injections.

**Methods:**

Luciferin delivered into the inner ears of GFAP-Luc transgenic mice reacted with luciferase in GFAP-expressing cells in the cochlear spiral ganglion, resulting in photon bioluminescence. We used the Xenogen IVIS® imaging system to measure how long photons continued to be emitted in the inner ear after TT or systemic injections of luciferin, and then compared the associated drug dynamics.

**Results:**

The response to TT and IP injections differed significantly. Photons were detected five minutes after TT injection, peaking at ∼20 minutes. By contrast, photons were first detected 30 minutes after i.p. injection. TT and i.p. drug delivery time differed considerably. With TT injections, photons were detected earlier than with IP injections. Photon bioluminescence also disappeared sooner. Delivery time varied with TT injections.

**Conclusions:**

We speculate that the drug might enter the Eustachian tube from the middle ear. We conclude that inner-ear drug concentration can be maintained longer if the two injection routes are combined. As the size of luciferin differs from that of therapeutics like dexamethasone, combining drugs with luciferin may advance our understanding of *in vivo* drug delivery dynamics in the inner ear.

## Introduction

Sensorineural hearing loss is mostly caused by inner ear disorders. Systemic injection of steroids is generally used in inner ear diseases, especially idiopathic sensorineural hearing loss. Recent studies show that an approach for drug delivery through the tympanic membrane to the round window (RW) into the inner ear is as effective as systemic injections [1]. However, there are a few studies examining the pharmacokinetics of inner ear drug delivery, and the time course remains elusive. Patients with hearing loss also exhibit a significant rate of RW obstruction, suggesting that drugs may not infiltrate the cochlea in these cases [2] [3]. When the RW is covered with connective tissue, we expect drug delivery into the inner ear can be facilitated.

Several animal experiment studies have been performed, but the inner ear has a very small volume of fluid, even when including both perilymph and endolymph (total 0.19 µl in mouse [4]). This makes reliable measurement difficult.

Microdialysis is an effective way for analyzing the cochlear fluid of animals. It has the advantage of making repeated measurements possible, allowing drug time course determination, preventing measurement artifacts arising through perilymph volume loss due to leaks, and lessens disturbance of perilymph due to the low volumes of drug recovery. However, there are several limitations associated with using microdialysis to analyze the pharmacokinetics of the inner: (1) technical difficulty of the measurement; (2) restricted locations available for measurement (i.e., basal turn of cochlea); (3) inability to quantify clearance values accurately for the inner ear; and (4) inability to measure the time course in the same animal. To solve these previously reported problems, we developed a new *in vivo* imaging technique and investigated whether RW obstructions block drug delivery into the inner ear.

## Materials and Methods

### GFAP-Luc transgenic mice

The transgenic GFAP-luc mice (FVB/N background; [5]) were obtained from Xenogen Corporation (Alameda, CA), and were backcrossed with CD1 (ICR, SLC Japan) for 5 or 6 generation. The mice contain a firefly luciferase gene expression cassette that is regulated by 12 kb of the murine GFAP promoter and the human β-globin intron 2 [5]. Luciferin delivered into the inner ear of these mice reacts with luciferase that is expressed in the GFAP-expressing cells in the cochlear nerve and spiral ganglion, and the resulting photons are detected by the camera. All experiments were approved by the Animal Care and Use Committee of Keio University (Permit Number: 08020) in accordance with the Guide for the Care and Use of Laboratory Animals (National Institute of Health, Bethesda,MD). All surgery was performed under ketamine and xylazine anesthesia, and all efforts were made to minimize suffering.

We divided the GFAP-Luc mice into three groups: intraperitoneal (i.p) injection group (N = 6), RW niche obstruction with transtympanic injection group (N = 3), and non-obstruction with transtympanic injection group (N = 8). The wild type mice group was transgene negative littermates (N = 2).

### Round window niche obstruction surgery

Adult GFAP-Luc mice were anesthetized with an intraperitoneal (i.p.) injection of ketamine (100 mg/kg) and xylazine (10 mg/kg). As previously described, an incision was made from the left post-auricular region to the submandibular region, and the tympanic bulla was exposed without damaging the facial nerve and vessels [6]. The bulla was perforated with a surgical needle, and the hole was expanded with forceps to approximately 2 mm diameter in order to cover over the RW with fascia (RW obstruction) without disturbing the auditory ossicular chain, mimicking clinical cases with RW false membrane. The hole in the cochlea and the opening in the tympanic bulla were sealed with carboxylate cement. Muscle and skin were sutured. It took approximately 20 min to complete this procedure. Left earlap was surgically removed to expose tympanic membrane for the injection of luciferin and to reduce the background.

### Transtympanic or intraperitoneal injections

Just prior to imaging, we injected 0.1 ml or 0.5 ml of luciferin substrate (150 mg/kg) directly onto the round window membrane or intraperitoneally, respectively. Transtympanic injection was performed securely under the surgical microscope.

### Statistics

Peak bioluminescence intensity and the time when the luminescence was maximum in individual groups (i.e., ip, TT injection and TT injection after round window closure) was examined by non-parametric t-test. All scores were averaged and analyzed with SPSS software 19.0 (Chicago, USA).

### Bioluminescence imaging

An IVIS spectrum and CCD optical macroscopic imaging system (Xenogen, Alameda, CA) was used for spatiotemporal detection of the luciferase - luciferin reaction. *In vivo* bioluminescent images were captured immediately after i.p. injection (0.5 ml) or after transtympanic injection (TT, 0.1 ml) of D-luciferin (D-(−)-2-(6-hydroxy-2-benzothiazolyl) thiazone-4-carboxylic acid). The field-of-view was set at 10 cm. The photon count was analyzed between 0 and 40 min after the i.p. and TT injections of luciferin substrate. The integration time was fixed at 5 min for each image. All images were analyzed with Living Image software (Xenogen, Alameda, CA). The optical signal intensity was expressed as photon flux (photon count), in units of photons/s/cm^2^/steradian. Each image was displayed as a pseudocolored photon-count image superimposed onto a grayscale anatomic image. To quantify the measured light, we defined regions of interest (ROI) over the cell-implanted area and examined all values in the same ROI.

### Immunohistochemistry

The animals were anesthetized with ketamine (100 mg/g) and xylazine (10 mg/g) and decapitated. The inner ear was removed and locally perfused with 4% paraformaldehyde in 0.1 M phosphate-buffered saline (PBS) followed by overnight fixation with the same fixative and wash with 0.1 M PBS. The otic capsules were embedded in paraffin and sectioned at 4 µm. For immunostaining, tissue sections were stained with anti-luciferase antibody (polyclonal goat, Promega) diluted at 1∶75 for 60 min at room temperature, followed by an anti-goat secondary antibody (Histofine® Simple Stain MAX-PO, Nichirei Bioscience; 414351).

## Results

### Characterization of the cochlea of GFAP-luc mouse

**Figure 1:**
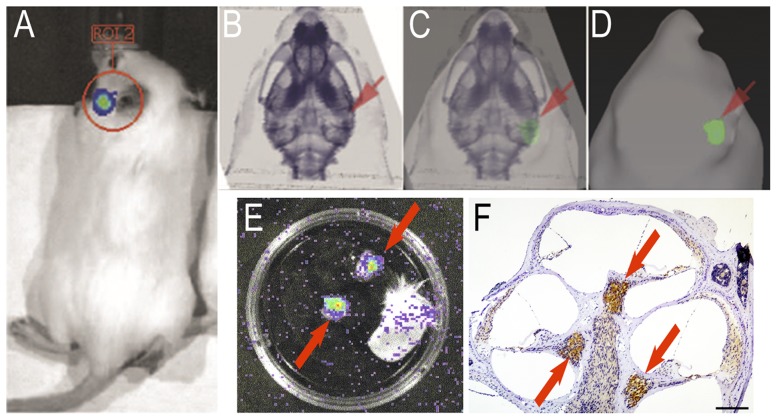
Strong bioluminescence around the ear of GFAP-luc derived from cochlear spiral ganglion. Previous bioimaging study with IVIS reported luminescence around the ear of GFAP-luc. We first reveal here that the signal results from spiral ganglion. (A) Signal observed after local injection of luciferin via tympanic membrane. (B–D) Bioluminescence image of mouse head overlaid with a corresponding micro-CT image. (B) Micro-CT image of the head. (C) Merged image of A and C. (D) Photon bioluminescence image of the head. Note the signal was overlapped with temporal bone and not with superficial skins. (E) Ex-vivo bioluminescence in a freshly dissected temporal bone. Strong signal in the cochleae was observed (arrows), while no signal was detected in the skin and ear cartridge (right). (F) Immnohistochemistry for luciferase in the cochlea showed the expression was high in spiral ganglion (arrows).

Previous bioimaging of GFAP-luc showed a signal around the ear [5]. We first investigated the origin of the signal. The signal was observed even after the local injection of luciferin thru tympanic membrane in the mouse of which earlap had been removed and the signal was seen over the temporal area (Fig. 1A). We then superimposed the biophotonic results over micro-CT image and the result clearly demonstrated that the signal was overlapped with temporal bone and not with superficial skins (Fig. 1B–D). We also dissected temporal bone of GFAP-luc and the luminescence of each part was measured: Strong signal was seen in the cochleae (Fig. 1E, arrows), while no signal was detected in the skin and ear cartridge (right). Collectively, we speculated that the signal in the ear area of the mice is derived from the cochlea. We then performed immnohistochemistry for luciferase in the cochlea, showing the expression was high in spiral ganglion (Fig. 1F, arrows). Together, we conclude that the bioluminescence in the area of GFAP-luc reflects the reaction of luciferase and luciferin in the cochlear spiral ganglion and thus we monitor the delivery of luciferin to the ganglion by measuring the biophotonic signals using IVIS.

### Bioimaging

**Figure 2:**
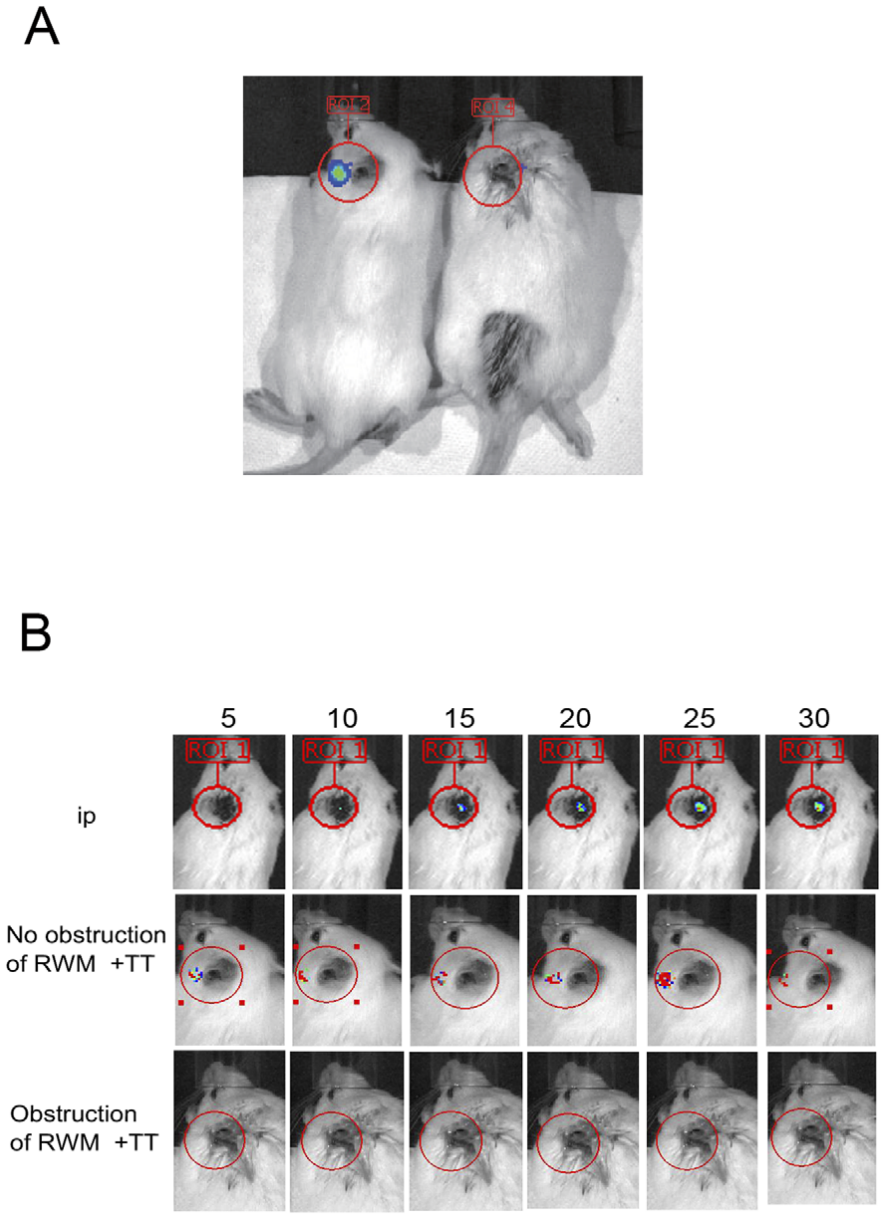
Photon bioluminescence in GFAP-luc mice. (A) Differences in photon bioluminescence of round windows with and without obstructions. The left panel shows photon bioluminescence in a mouse's left ear, in which the round window has been obstructed. The right panel shows the lack of bioluminescence in the mouse's right ear, in which the round window remains intact. (B) Time course showing photon bioluminescence in ears of mice with ip, with and without round window obstruction. ip;intraperitoneal injection, RWM; round window membrane.

In the TT injection group of mice, photon bioluminescence was observed five minutes after injection, reaching a peak 15–20 minutes after injection (Fig. 2B). Bioluminescence intensity gradually faded thereafter. In the i.p. injection group of mice, detectable bioluminescence appeared much later than that in the TT injection group. As expected, no bioluminescence was observed in the RW obstruction group (negative control). The lack of reaction in the negative control indicated that luciferase did not reach the inner ear.

### Time curves

**Figure 3:**
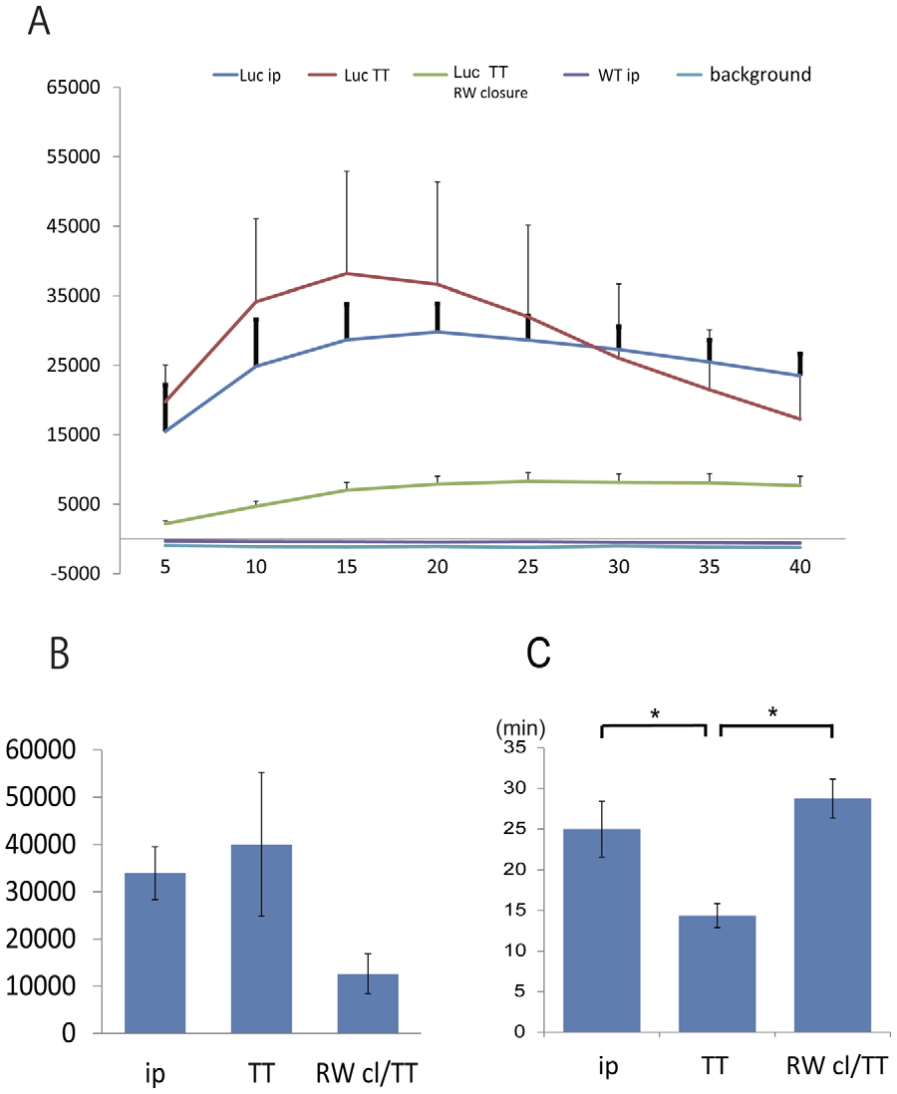
Time course of photon bioluminescence. (A) Time course of photon bioluminescence in five groups of animals: Luc ip, luciferase-expressing mice receiving intraperitoneal injections; Luc TT, luciferase-expressing mice receiving transtympanic injections; Luc TT RW closure, luciferase-expressing mice that received transtympanic injections after round window membrane obstruction; WT ip, wild-type mice receiving intraperitoneal injections. Background, background (no photons). (B) Bar graphs showing the highest photon counts by region. ip, intraperitoneal injection; TT, transtympanic injection; RW cl/TT, transtympanic injection after round window membrane obstruction. (C) Delivery time into GFAP-expressing cells of inner ear. Average time when the signal was maximum in each group was shown (mean ± SEM). Same conventions as in Fig. 2. *p<0.05.

**Figure 4:**
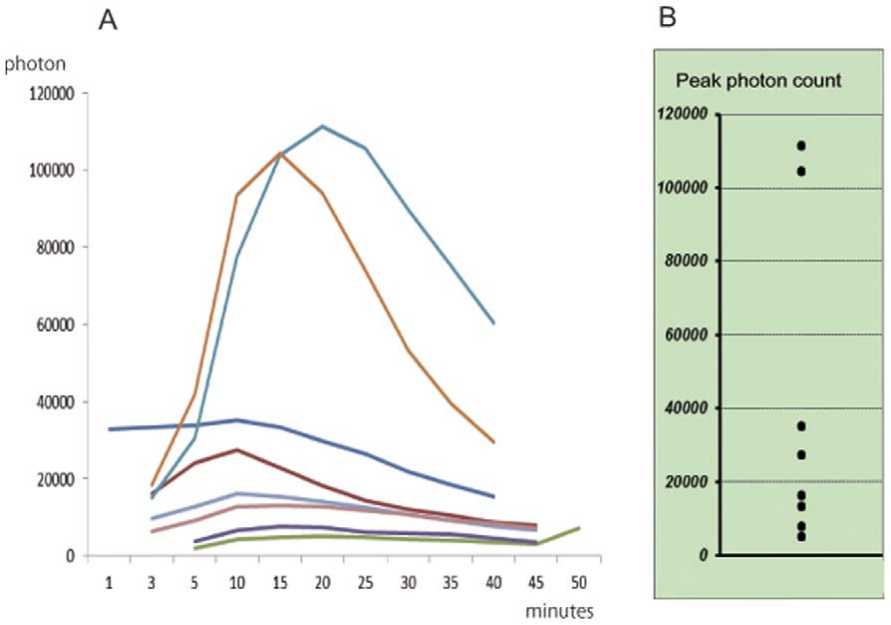
Quantification of photons in different groups of mice with TT injections. (A) Time course of photon bioluminescence for each group of mice. (B) Peak photon counts.

Trends of bioluminescence in GFAP-luc mouse and wild type control in the individual groups were examined (Fig. 3A, mean ± SD). Peak bioluminescence intensity in the TT injection group was reached sooner after injection than in the i.p. injection group (Fig. 3C, mean ± SEM p<0.05). Time course of the photon emission in the individual animals and their peak of TT injection group are in Fig. 4A and 4B, respectably.

## Discussion

We found that RW niche obstructions, such as false membranes or fibrous connective tissue membranes, are critical for drug delivery into the inner ear. Therefore, it is very important to examine the RW before injecting drugs into the inner ear [3].

The present study also assessed the time needed for TT injections of drug delivery system (DDS) to reach the inner ear and traced DDS distribution in the inner ear. A previous report demonstrated that one hour after TT injection of methylprednisolone, dexamethasone, and hydrocortisone, drug concentration in the perilymphatic space becomes elevated [7]. However, a few studies report shorter drug elevation times after injection [8], [9], [10]. In this study, we detected bioluminescence in the inner ear earlier than expected; five minutes after TT injection of luciferin. A study in guinea pigs showed that significantly higher dexamethasone levels were detected in the inner ear just 30 minutes after its transtympanic administration, gradually dropping to zero after 6 hours [9]. The time differences may be due to differences in the sizes of animals (guinea pigs vs. mice), and/or due to differences in drug metabolism and drug molecular size (steroid vs. luciferin).

We observed variations in bioluminescence intensity among animals in the TT group. Individual variations between animals, especially in the local injection group, have been reported previously [7], [8]. We speculated that the injected luciferin goes to the Eustachian tube of animals.

In Chandrasekhar et al., drug concentration in perilymph was 5.52±2.80 µg/dL after intravenous (i.v.) injection of 0.45 mg/kg dexamethasone, but 13.2±10.6 µg/dL after location injection of 10 mg/mL dexamethasone. Although the dose of the local injection was approximately 10 times higher than that of the systemic injection, drug concentration in perilymph was nearly two times lower (5.52±2.80 µg/dL vs. 13.2±10.6 µg/dL) [8]. In the present study, although the dose of the systemic injections was five times higher than that of TT injections, drug concentration in perilymph was almost the same or slightly higher.

Here, we were able to observe, in real time, drug delivery and drug distribution in animals *in vivo*, enabling us to determine the time course of drug delivery. One limitation of this study, however, is that we investigated the delivery of only luciferin into the inner ear, not drugs commonly used to treat inner ear disorders. Differences in the molecular weights of luciferin and steroids could affect delivery and diffusion time. One future application of this system is drug tagging, in which a drug linked to luciferin can be traced. This drug-tagging system may help physicians determine which drug can be delivered into which portion of the inner ear.
